# Popularity and performance of bioinformatics software: the case of gene set analysis

**DOI:** 10.1186/s12859-021-04124-5

**Published:** 2021-04-15

**Authors:** Chengshu Xie, Shaurya Jauhari, Antonio Mora

**Affiliations:** Joint School of Life Sciences, Guangzhou Medical University and Guangzhou Institutes of Biomedicine and Health - Chinese Academy of Sciences, Guangzhou, China

**Keywords:** Pathway analysis, Gene set analysis, Benchmark, GSEA

## Abstract

**Background:**

Gene Set Analysis (GSA) is arguably the method of choice for the functional interpretation of *omics* results. The following paper explores the popularity and the performance of all the GSA methodologies and software published during the 20 years since its inception. "Popularity" is estimated according to each paper's citation counts, while "performance" is based on a comprehensive evaluation of the validation strategies used by papers in the field, as well as the consolidated results from the existing benchmark studies.

**Results:**

Regarding popularity, data is collected into an online open database ("GSARefDB") which allows browsing bibliographic and method-descriptive information from 503 GSA paper references; regarding performance, we introduce a repository of jupyter workflows and shiny apps for automated benchmarking of GSA methods (“GSA-BenchmarKING”). After comparing popularity *versus* performance, results show discrepancies between the most popular and the best performing GSA methods.

**Conclusions:**

The above-mentioned results call our attention towards the nature of the tool selection procedures followed by researchers and raise doubts regarding the quality of the functional interpretation of biological datasets in current biomedical studies. Suggestions for the future of the functional interpretation field are made, including strategies for education and discussion of GSA tools, better validation and benchmarking practices, reproducibility, and functional re-analysis of previously reported data.

**Supplementary Information:**

The online version contains supplementary material available at 10.1186/s12859-021-04124-5.

## Background

Bioinformatics method and software selection is an important problem in biomedical research, due to the possible consequences of choosing the wrong methods among the existing myriad of computational methods and software available. Errors in software selection may include the use of outdated or suboptimal methods (or reference databases) or misunderstanding the parameters and assumptions behind the chosen methods. Such errors may affect the conclusions of the entire research project and nullify the efforts made on the rest of the experimental and computational pipeline [1].

The following paper discusses two main factors that motivate researchers to make method or software choices, that is, the *popularity* (defined as the perceived frequency of use of a tool among members of the community) and the *performance* (defined as a quantitative quality indicator measured and compared to alternative tools). The study is focused on the field of “gene set analysis” (GSA), where the popularity and performance of bioinformatics software show discrepancies, and therefore the question appears whether biomedical sciences have been using the best available GSA methods or not.

**Fig. 1:**
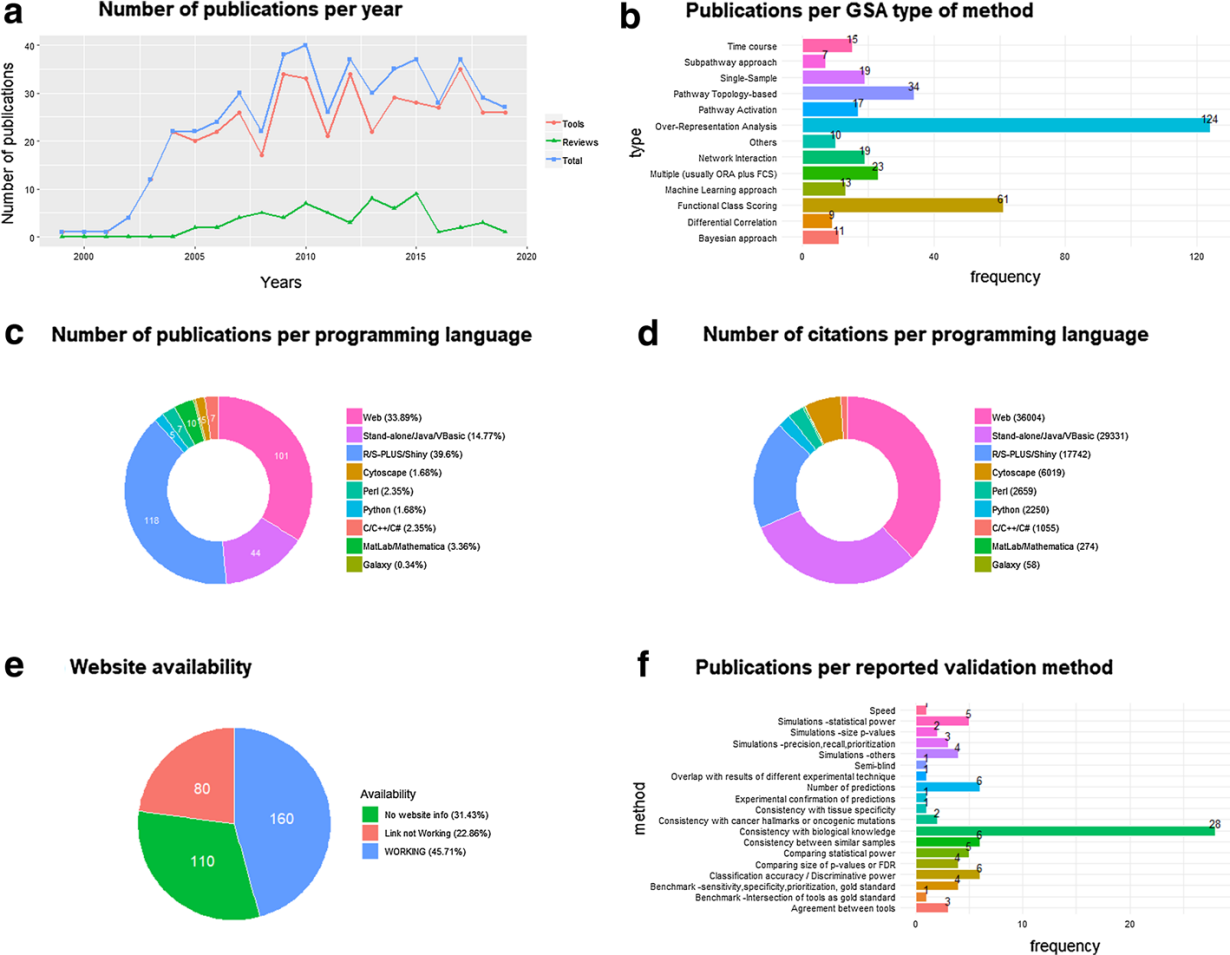
Statistics from GSARefDB v.1.0 (for more recent statistics, visit our website: https://gsa-central.github.io/gsarefdb.html). **a** Number of GSA publications per year. **b** Number of publications per type of GSA method. **c** Number of publications per used programming language. **d** Number of citations per used programming language. **e** Website availability. **f** Number of publications per reported validation method

GSA is arguably the most common procedure for functional interpretation of *omics* data, and, for the purposes of this paper, we define it as the comparison of a query gene set (a list or a rank of differentially expressed genes, for example) to a reference database, using a particular statistical method, in order to interpret it as a rank of significant pathways, functionally related gene sets, or ontology terms. Such definition includes the categories that have been traditionally called 'gene set analysis', 'pathway analysis', 'ontology analysis', and 'enrichment analysis'. All GSA methods have a common goal, which is the interpretation of biomolecular data in terms of annotated gene sets, while they differ depending on characteristics of the computational approach (for more details, see 'Methods' section, as well as Fig. 1 of reference [2]). GSA has arrived to 20 years of existence since the original paper of Tavazoie et al*.* [3], and many statistical methods and software tools have been developed during this time. A popular review paper listed 68 GSA tools [4], while a second review reported an additional 33 tools [5], and a third one, 22 tools [6]. We have built the most comprehensive list of references to date (503 papers), and we have quantified each paper’s influence according to their current number of citations (see Additional file 1 and reference [7]). The most common GSA methods include Over-Representation Analysis (ORA), such as DAVID [8], Functional Class Scoring (FCS), such as GSEA [9], and Pathway-Topology-based (PT) methods, such as SPIA [10], which have all been extensively reviewed. In order to know more about them, the reader may consult any of the 62 published reviews documented in Additional file 1. Recently, we have also reviewed other types of GSA methods [2].

The first part of the analysis is a study on GSA method and software popularity based on a comprehensive database of 503 papers for all methods, tools, platforms, reviews, and benchmarks of the GSA field, collectively cited 134,222 times between 1999 and 2019, including their popularity indicators and other relevant characteristics. The second part is a study on performance based on the validation procedures reported in the papers introducing new methods together with all the existing independent benchmark studies in the above-mentioned database. Instead of recommending one single GSA method, we focus on discussing better benchmarking practices and generating benchmarking tools that follow such practices. Together, both parts of the study allow us to compare the popularity and performance of GSA tools but also to explore possible explanations for the popularity phenomenon and the problems that limit the execution and adoption of independent performance studies. In the end, some practices are suggested to guarantee that bioinformatics software selection is guided by the most appropriate metrics.

## Results

### Popularity

The number of paper citations has been used as a simple (yet imperfect) measure of a GSA method’s popularity. 350 references to GSA papers of methods, software or platforms have been collected (Additional file 1: Tab 1), as well as 91 references to GSA papers for non-mRNA *omics* tools (Additional file 1: Tabs 3, 4), and 62 GSA reviews or benchmark studies (Additional file 1: Tabs 2, 3, 4), which have been organized into a manually curated open database (GSARefDB). GSARefDB can be either downloaded or accessed through a shiny interface at: https://gsa-central.github.io/gsarefdb.html. Figure 1 summarizes some relevant information from the database (GSARefDB v.1.0). The citation count shows that the most influential GSA method in history is Gene Set Enrichment Analysis (GSEA), published in 2003, with more than twice as many citations as its follower, the ORA platform called DAVID (17,877 versus 7500 citations). In general, the database shows that the field contains a few extremely popular papers and many papers with low popularity. Figure 1b shows that the list of tools is mainly composed of ORA and FCS methods, while the newer and less known PT and Network Interaction (NI) methods are less common (and generally found at the bottom of the popularity rank).

It could be hypothesized that the popularity of a GSA tool does not always depend on being the best for that particular project, and it could be related to variables such as its user-friendliness instead. The database allows us to compute citations-per-programming-language, which we use as an approximation to friendliness. Figure 1c shows that the majority of the GSA papers correspond to R tools, but, in spite of that, Fig. 1d shows that most of the citations correspond to web platforms followed by stand-alone applications, which are friendlier to users. Worth mentioning, the last column of the database shows that there are a large number of tools that are not maintained anymore and broken web links to tools or databases, which makes their evaluation impossible. This phenomenon is a common bioinformatics problem that has also been reported elsewhere [11]. Figure 1e shows that one-third of the reported links in GSA papers are now broken links.

Besides ranking papers according to their all-time popularity, a current-popularity rank was also built (Additional file 1: Tab 5). To accomplish that, a version of the database generated in May-2018 was compared to a version of the database generated in April-2019. The current-popularity rank revealed the same trends than the overall-rank. GSEA is still, by far, the most popular method. The other tools that are being currently cited are clusterProfiler [12], Enrichr [13], GOseq [14], DAVID [8], and ClueGO [15], followed by GOrilla, KOBAS, BiNGO, ToppGene, GSVA, WEGO, agriGO, and WebGestalt. ORA and FCS methods are still the most popular ones, with 3534 combined citations for all ORA methods and 2185 citations for all FCS methods, while PT and NI methods have 111 and 50 combined citations respectively. In contrast, single-sample methods have 278 combined citations, while time-course methods have 67. Regarding reviews, an extremely popular paper from 2009 [4] is still the currently most popular, even though it doesn’t take into account the achievements of the last ten years.

### Performance

The subject of validation of bioinformatics software deserves more attention [16]. A review of the scientific validation approaches followed by the top 153 GSA tool papers in the database (Additional file 1: Tab 6) found multiple validation strategies that were classified into 19 categories. 61 out of the 153 papers include a validation procedure, and the most commonly found validation strategy is “Consistency with biological knowledge”, defined as the fact that our method’s results explain the knowledge in the field better than the rival methods (which is commonly accomplished through a literature search). Other common strategies (though less common) are the comparisons of the number of hits, classification accuracy, and consistency of results between similar samples. Important strategies, such as comparing statistical power, benchmark studies, and simulations, are less used. The least used strategies include experimental confirmation of predictions and semi-blind procedures where a person collects samples and another person applies the tool to guess tissue or condition. Our results have been summarized in Fig. 1f and Additional file 1: Tab 6. We can see that the frequency of use of the above-mentioned validation strategies is inversely proportional to their reliability. For example, commonly used strategies such as “consistency with biological knowledge” can be subjective, and comparing the number of hits of our method with other methods on a Venn diagram [17] is a measure of agreement, not truth. On the other hand, the least used strategies, such as experimental confirmation or benchmark and simulation studies, are the better alternatives.

The next step of our performance study was a review of all the independent benchmark and simulation studies existing in the GSA field, whose references are collected in the Additional file 1: Tab 2. Table 1 summarizes 10 benchmark studies of GSA methods, with different sizes, scopes, and method recommendations. A detailed description and discussion of each of the benchmarking studies can be found in the Additional file 2. The sizes of all benchmarking studies are small when compared to the amount of existing methods that we have mentioned before, while their lists of best performing methods show little overlap. Only a few methods are mentioned as best performers in more than one study, including ORA methods (such as hypergeometric) [3], FCS methods (such as PADOG) [18], SS methods (such as PLAGE) [19], and PT methods (such as SPIA/ROntoTools) [20].

**Table 1:** Benchmarking studies of GSA methods

References	Scope	Size	Criteria	Best performing methods
Naeem et al. [21]	ORA and FCS methods	14 methods	Method’s AUC (evaluated by predicting targets of TFs and miRNAs)	ANOVA, Z-SCORE, and Wilcoxon’s rank sum (WRS)
Tarca et al. [22]	ORA, FCS, and SS methods	16 methods	Prioritization, Sensitivity, and FPR	GLOBALTEST and PLAGE (sensitivity), PADOG and ORA (prioritization), and CAMERA (FPR). Author’s general recommendation: PLAGE, GLOBALTEST, and PADOG
Bayerlova et al. [23]	ORA, FCS, and PT methods	7 methods	Sensitivity and prioritization (for benchmark), and Sensitivity, specificity, and accuracy (for simulations of pathway overlap)	For benchmark: CePaGSA (sensitivity) and PathNet (prioritization). For simulation -original pathways: CePAGSA (sensitivity), WRS (specificity), and WRS (accuracy). For simulation -non-overlapping pathways: KS (sensitivity), and SPIA, CePaORA, CePaGSA, and PathNet (specificity and accuracy)
Jaakkola et al. [24]	ORA, FCS, and PT methods	5 methods	Consistency of significant pathways between datasets, and Sensitivity	SPIA and CePaORA (consistency), and SPIA, CePaORA, and NetGSA (sensitivity). Author’s general recommendation: SPIA
De Meyer et al. [25]	ORA, FCS, and NI methods	4 methods	Prioritization, Sensitivity, and Specificity	PADOG (specificity) and BinoX (sensitivity)
Lim et al. [26]	SS/Pathway-activity methods	13 methods	Classification performance, preservation of data structure, robustness to noise, and reproducibility between pathway databases	ESEA, Pathifier, SAS, and PADOG (classification tasks), Pathifier and PLAGE (data structure), ssGSEA (robustness), and individPath, Pathifier, and SAS (reproducibility). Author’s general recommendation: Pathifier, SAS, and individPath
Nguyen et al. [27]	ORA, FCS, and PT methods	13 methods	In order of importance: Number of biased pathways, Prioritization, Method’s AUC, and sensitivity (evaluated using both disease target pathways and KO data)	GSEA (bias), PADOG (prioritization), ROntoTools (AUC), and CePaGSA (p-values). Author’s general recommendation: ROntoTools
Ma et al. [28]	FCS, PT, and NI methods	9 methods	Ranking of empirical powers	DEGraph, followed by PathNet and NetGSA
Zyla et al. [29]	ORA, FCS, and SS methods	9 methods	Sensitivity, FPR, prioritization, computational time, and reproducibility	PLAGE (sensitivity), ORA and PADOG (specificity/FPR), PADOG (prioritization), and CERNO (reproducibility)
Geistlinger et al. [30]	ORA, FCS, and SS methods	10 methods	Sensitivity, computational time, and phenotype relevance score	Author’s general recommendation: ROAST and GSVA (for self-contained hypothesis). ORA and PADOG (for competitive hypothesis)

Ten benchmark studies from 2012 to 2020, showing a plurality of scopes, sizes, and method recommendations. Details on each study can be found in Additional file 2

ORA, over-representation analysis; FCS, functional class scoring; PT, pathway topology-based; SS, single-sample; NI, network interaction

It is crucial to notice that the previous studies just covered a small fraction of the entire universe of GSA methods. Also, that there is little overlap between the sets of methods included in different studies, but nevertheless we can still find inconsistencies between results, such as the GSVA, Pathifier, and hypergeometric methods, which are reported both as best performer and as poor performer in different benchmarks. From all the above-mentioned high-performance tools, only “ORA” appears in the top 20 currently most popular tools (Additional file 1: Tab 5), suggesting that there is a divorce between popularity and performance (see also Additional file 2: Table 2).

### Popularity versus performance

In general terms, it is evident that performance studies are still few, small, inconsistent, and dependent on the quality of the benchmarks; however, they tend to recommend tools different to the popular and friendly ones.

GSEA is one of the most important landmarks in GSA history and the most striking example of the contradictions between popularity and performance metrics of GSA tools. With one exception, none of the recent benchmarks under analysis has reported GSEA to be the best performing method; however, GSEA is still both the all-time and the currently most used tool. Besides that, numerous methods (most of them not included in the previous benchmarks) report that they specifically outperform GSEA in at least one of many possible ways; such methods are highlighted on Additional file 1: Tab 7 with the tag "COMPARED". In addition, there have been plenty of developments to the GSEA method itself; for example, alternative functions to score the gene set, alternative options to the permutation step to finding p-values, or using GSEA as part of an extended method (see methods at Additional file 1: Tab 7, with the tag "PART OF METHOD"), which reportedly outperform the original GSEA. We have identified a total of 79 papers in those categories (Additional file 1: Tab 7). However, in spite of that, GSEA's overwhelming popularity compared to any other method would suggest otherwise. We can also verify that, with the exception of ORA, most of the above-mentioned high performing methods tend to occupy lower places in the current popularity ranking (Additional file 2: Table 2).

It is hard to estimate how many conclusions from how many articles should be rectified if we rigorously apply our current knowledge on GSA and use the best performing methods in every paper that ever used GSA. However, taking into account that our database (Additional file 1) registers 503 GSA software and tool papers that have been collectively cited 134,222 times, there is a valid concern regarding the need to pay more attention to both the method performance studies and the performance-based tool selection in GSA. Otherwise, the last step of the *omics* data analysis process may undermine the efforts on the rest of the pipeline.

### Performance tools

The previous observation regarding the difference between the most popular and the best performing GSA methods could be incorrect, given that existing benchmarks have followed different methodologies and the proper way to benchmark GSA methods is a discussion in itself. Therefore, we have also reviewed and discussed the existing benchmarking and simulation strategies, in order to extract a few ideas regarding good benchmark practices.

Rigorous benchmark studies are not a straightforward task, given that there is no accepted gold standard to be used in a comparison of GSA tools [25]. Reviewed here are three strategies to build such a gold standard. The first strategy is to apply several different enrichment tools to the same gene sets and use the intersection of the results as a gold-standard. One example of this is EnrichNet [31], which obtains a “set of high confidence benchmark pathways” as the intersection between the top 100 ranks generated by the SAM-GS and GAGE methods. Such a procedure is questionable, as the chosen methods are far from being considered the best (as seen before), and the procedure is more related to agreement than truth. The second strategy is the use of disease datasets clearly associated with one pathway as a gold-standard. For example, Tarca et al*.* [22] compiled 42 microarray datasets with both healthy and disease-associated samples, where the disease was associated with a KEGG pathway (and, therefore, such “target pathway” should be found significant) [22]. The third strategy is to leverage knowledge on gene regulation effects. Some authors have used gene sets built from the known targets of specific transcription factors and miRNAs, and then try to predict the changes after over-expression or deletion of such regulators [21], while some others have used datasets with mouse KOs: Pathways containing the KO gene were considered as target pathways, while the others were considered as negatives [27]. Geistlinger et al*.* [30] have recently introduced a modification of such approach in which they not only look at “target pathways” matching the original diseases, but instead create a “gene set relevance ranking” for each disease. To build such rankings, the authors used MalaCards gene scores for disease relevance, which are based on both experimental and bibliometric evidence; then, they used the gene scores to build combined gene set relevance scores for all GO and KEGG terms (using the GeneAnalytics tool). As a result, instead of benchmarking against some “target pathways”, the benchmark is done against a “pathway relevance ranking” per disease. Table 2 assesses different performance measurement approaches according to their objectivity, reproducibility, and scalability.

**Table 2:** Comparison of performance criteria

Criteria	Objectivity	Reproducibility	Scalability	Drawbacks
Tool agreement	Low	High	High	Subjective
Consistency between similar samples	Low	High	High	Subjective
Consistency with biological knowledge (Literature search)	Low	Low	Low	Subjective
Benchmark (target pathways as gold standard)	High	High	High	Centered on true positives
Benchmark (pathway relevance ranking as gold standard)	High	High	High	–
Benchmark (KO/perturbation data as gold standard)	High	High	High	–
Simulations	Low	Low	High	Human-designed datasets may be unrealistic

Objectivity, Reproducibility, and Scalability of the main performance criteria. Objectivity refers to the results not depending on human interpretation. Reproducibility refers to any researcher being able to find the same results by following the same procedures. Scalability refers to the possibility of easily applying the procedure to an increasingly higher number of methods and datasets

A second issue is the selection of the benchmark metrics that quantitatively determine who is the best performer. Well-known metrics such as sensitivity, specificity, precision, or the area under the ROC curve, have been traditionally used. Tarca et al*.* introduced the use of sensitivity, specificity, and “prioritization”, together with False Positive Rate (FPR), where prioritization is a concept related to the rank of the target pathways for a given method. Zyla et al*.* have recently extended Tarca's work and recommended using a set of five different metrics: Sensitivity, False positive rate (FPR), Prioritization, Computational time, and Reproducibility. In their approach, reproducibility assigned a high score to methods that showed similar results for different datasets coming from the same condition/disease and the same technical platform, but different authors/labs [29]. The chosen metrics are fundamental because no method is the best under all metrics and every user should start by selecting a method according to the goals of their study, which may need a very sensitive method, a very specific method, or any other of the above-mentioned properties. As a consequence, benchmark studies must be clear regarding the metrics under which they are ranking the method performance [22], and their set of metrics should at least include the previously mentioned ones. Also, it makes no sense to declare success because a new method has better sensitivity than other methods that are known for a poor sensitivity; therefore, it is a logical consequence that any new methods must compare their sensitivity to sensitive methods, their specificity to specific methods, and so on.

A third issue is that validation procedures should not allow the authors to subjectively choose the methods that the authors want to compare to their new method, as it would be possible for the authors to choose those methods that they can outperform. One alternative to that is using well-established, independent, and comprehensive benchmark studies as a reference; then, when a new method appears, validation should be done by comparison to the top methods from such independent benchmarks. This practice is not common; as an exception, the authors of LEGO [32] explicitly use the top 5 methods in Tarca et al.'s benchmark [22].

As a final thought, ensembles of methods have been suggested to give better results than any of the single methods [33]. That implies that benchmark studies should not be limited to single tools but include comparisons to ensembles of tools as well. This approach has been followed by at least one benchmark study [21].

The previous analysis makes us think that future GSA benchmarks should include the most recent developments on benchmark theory, as well as be performed on more GSA methods, in order to extract more meaningful conclusions. In order to do this, we have also created a benchmarking platform, “GSA-BenchmarKING”, which is a repository of apps/workflows/pipelines that follow the above-mentioned good benchmarking practices and allow benchmarking of GSA software in an easy and automated way. Currently, GSA-BenchmarKING contains Jupyter notebooks with full workflows for benchmarking GSA methods, and shiny apps that allow benchmarking with the click of a few buttons. The initial benchmarking tools added to the repository were created by our students as an example, following the previous guidelines, but they are open software and can be permanently improved. The initial tools are focused on two types of methods: Single-sample GSA (such as GSVA, Pathifier, SSGSEA, PLAGE, ZSCORE) and genomic-region GSA (such as GREAT, ChIP-Enrich, BroadEnrich, Enrichr, Seq2Pathways). All apps allow the user to define different gold standard datasets (or use ours), select the GSA methods to compare, and select the comparison metrics to plot. In addition, it is possible to keep adding more GSA methods to the app with the help of the community, as each app includes instructions for programmers interested in adding new methods. Given the large amount of GSA methods, more benchmarking tools are needed and welcomed. GSA-BenchmarKING can be accessed at: https://gsa-central.github.io/benchmarKING.html.

## Discussion

The GSARefDB has been used to make some initial data exploration on the relationship between popularity and performance of GSA tools. Besides the observations highlighted in this article, such a database can also be the source for more in-depth research. For example, some limitations of our approach include (i) that some papers describe more than one method or platform, and (ii) that some methods are only cited for comparison purposes. We observe that such problems are the exception and not the rule, but future studies might want to take them into account and GSARefDB can still be used as a data source for it.

In general terms, it is evident that performance studies are still few, small, inconsistent, and dependent on the quality of the benchmarks; however, they tend to recommend tools different to the popular and friendly ones. Among the possible reasons for the discrepancies between popularity and performance, it has been suggested that software selection may not be entirely related to performance but to factors such as a preference for user-friendly platforms or user-friendly concepts or plots (for example, GSEA’s “enrichment plot”). Also, due to the fact that the objective evaluation of the performance of the different GSA methods is a complicated and time-consuming issue, or to the fact that new methods need more time to be accepted. In network analysis, popularity can be explained by the “rich-gets-richer” (popular-gets-more-popular) effect. Using concepts of the “consumer behavior” field, software selection can be studied as the choice of a popular brand, that is, variables such as: Confidence in experience (the respect to the researcher/institution associated to the software), social acceptance and personal image (following the software that everyone else is using), or consumer loyalty (after some time, we are attached to our software and not interested to change) [34]. In a recent book [35], Barabasi has suggested that popularity and quality usually go together in situations where performance is clearly measurable. Otherwise, popularity can't be equated to quality. Our study agrees with such an idea. We have found that the most popular GSA software is different from the best performing GSA software, and that thorough performance evaluation is still a pending assignment for bioinformaticians.

However, in the specific case of GSA, there is a complementary explanatory hypothesis: Besides all the progress in GSA theory leading to better-performing methods, at the end of the pipeline, the users usually extract the evidence that they consider relevant from the resulting rank of gene sets, choosing the pathways or GO terms of their interest and ignoring the rest of the ranking, and, therefore, any differences between methods regarding lower p-values, prioritization order, and so on, become less important. It is said that in the step of gene set selection from the final gene set ranking, researchers bring "context" to the results but, this way, their subjectivity may be projected to the study. One way out of this is to stimulate the research on “context-based GSA”. For example, a recent effort called contextTRAP [36] combines an impact score (from pathway analysis) with a context score (from text mining information that supports that the pathway is relevant to the context of the experiment). Bayesian approximations to GSA using text mining data as context need to be more developed, as any other method that studies the final gene set ranking as a whole.

## Conclusions

Given that popular methods are not necessarily the best, bioinformatics software users should not only be guided by popularity but mainly by performance studies. However, performance studies, in time, must be guided by the general guidelines that we have discussed, that is, researchers should only follow the most convincing benchmark procedures. Such strict recommendations are problematic because performance studies are few, low-coverage, and have a variable quality; therefore, we need more open tools to dynamically review popularity and performance of bioinformatics software, such as those introduced here.

Based on the previous results and discussion, we argue that the functional interpretation field would benefit from:

1. Having more information and discussion regarding the nature and scope of the existing functional interpretation methods of *omics* data, as well as more teaching of new and sophisticated methods in bioinformatics courses, more guidelines for selection of a tool, and more popularization of the functional interpretation shortcomings. Also, requesting deeper discussions of the selection of GSA methods for all biomedical papers.
2. The permanent evaluation of GSA methods, including better gold standards, more and more comprehensive comparison studies, and better benchmarking practices. The fact that two prestigious computational biology journals have recently created a special edition and a collection specialized on benchmarks are welcome steps in that direction [37, 38].
3. Paying attention to reproducibility and offering open code in code-sharing platforms (such as Github), containers with the specific software and library versions used in their work (such as Docker), notebooks (such as RStudio and Jupyter) including scripts with a detailed explanation of their methods, and other strategies to allow reproducibility.
4. The creation of a culture of functional re-analysis of existing data using new GSA methods, as well as the computational tools to functionally re-analyze existing *omics* datasets in a streamlined manner.
5. More rigorous validation procedures for GSA tools. Also, bioinformaticians should get as much training on scientific validation methods and tools as they get on using and building bioinformatics tools.

Proper tool selection is fundamental for generating high-quality results in all scientific fields. This paper suggests that tool performance and tool selection studies, via the popularity-performance evaluation based on an exhaustive reference database, is a methodology that should be followed up, to keep track of the evolution of the tool selection issues in a scientific field. We have also introduced examples of popularity and performance-measuring software that could help making such studies easier. The reader is here invited to keep following our work for the GSA field at: https://gsa-central.github.io/gsarefdb.html and https://gsa-central.github.io/benchmarKING.html.

## Methods

### Definitions

The concepts involved in this study have been defined in several different ways in the literature. For example, the field under study has been called "Pathway Analysis" [5], "Enrichment analysis" [23], "Gene Set Analysis" [2], "Functional enrichment analysis" [12], “Gene-annotation enrichment analysis” [4], and other terms, by different authors. At the same time, the term "Gene Set Analysis" has been used to describe the entire field [2], or just the group of ORA and FCS methods (in opposition to methods including pathway or network topology) [23], or even one specific tool [39]. Finally, the term “Pathway Analysis” has also been used to both describe the entire field [5] or just the group of methods that include pathway topology [23]. For this reason, we have added the following summary of the definitions used in this study for the above-mentioned and other relevant terms.

*Gene Set Analysis (GSA)*: GSA methods have been defined as a group of "methods that aim to identify the pathways that are significantly impacted in a condition under study" [6] or as "tests which aim to detect pathways significantly enriched between two experimental conditions" [23]. More specifically, GSA is an annotation-based approach that statistically compares experimental results to an annotated database in order to transform gene-level results into gene-set-level results. For example, a query gene set (a list of differentially expressed genes, or a rank of all gene’s fold change) is mapped to a gene set reference database, using a particular statistical method, in order to explain the experimental results as a rank of significantly impacted pathways, functionally related gene sets, or ontology terms.

*Over-Representation Analysis (ORA)*: A subset of GSA methods based on comparing a list of query genes (for example, up- or down-regulated genes) to a list of genes in a class or gene set, using a statistical test that detects over-representation. ORA "statistically evaluates the fraction of genes in a particular pathway found among the set of genes showing changes in expression" [5].

*Functional Class Scoring (FCS)*: A subset of GSA methods in which the values of a gene-level statistic for all genes in the experiment are aggregated into a gene-set-level statistics [5], and gene set enrichment is computed in terms of the significance of such gene-set-level statistic. FCS methods start from a quantitative ranking of the gene-level statistic for all genes under analysis (in contrast to ORA, which only uses a list of differentially expressed genes). Some popular FCS methods find if the relative position of a gene set in the ranking of all genes is shifted to the top or the bottom of the ranking. For example, the WRS test compares the distribution of ranks of the genes in a gene set to the distribution of ranks of the genes in the complement to the gene set, while the KS test compares the ranks of genes in a gene set to a uniform distribution [23].

*Pathway-Topology-based (PT)*: A subset of GSA methods that weights enrichment scores according to the position of a gene in a pathway. Only applies to pathway data and not to other types of gene sets.

*Network Interaction (NI)*: A subset of GSA methods that not only includes the given gene sets but also the gene products that interact with the members of such gene sets when located on top of an interaction or functional annotation network.

*Popularity*: The frequency of use of a method or tool among members of a community.

*Performance*: The value of a quantitative property (when compared to alternative methods or tools) that measures the agreement between the method's output and either empirical data, simulated data, or the output of another method.

*Benchmark study*: A systematic comparison between computational methods, in which all of them are applied to a gold standard dataset and the success of their gene set predictions are summarized in terms of quantitative metrics (such as sensitivity, specificity, and others).

*Simulation study*: A systematic comparison of computational methods based on building artificial datasets that possess the properties that we specify for them.

*Gold standard*: A "perfect gold standard" would be an error-free dataset that can be used as a synonym for truth (in our case, an *omics* dataset associated to a *true* ranking of pathways); however, in practice, we are limited to use "imperfect" or "alloyed gold standards", which are datasets confidently linked to the truth, but not necessarily datasets lacking error [40].

### Construction of the database

The Gene Set Analysis Reference Database (GSARefDB) was built from the following sources:

1. Google and PubMed searches of keywords such as: "Pathway analysis", "Gene set analysis", and "Functional enrichment" (approx. 10% of the records).
2. Cross-references from all the collected papers and reviews (approx. 50% of the records).
3. Email alerts received from NCBI and selected journals (approx. 40% of the records).

**Fig. 2:**
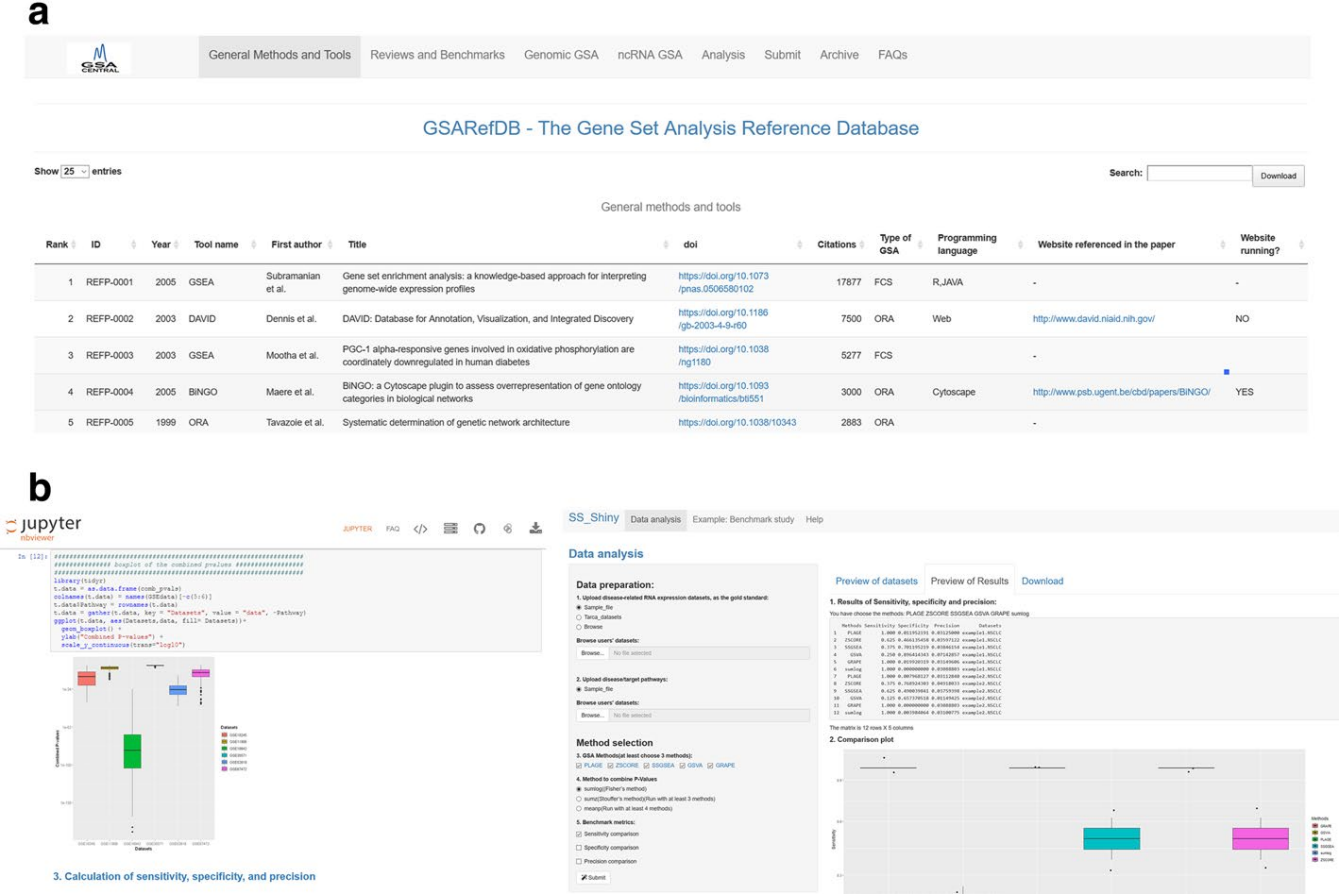
Screenshots of our tools for popularity and performance analysis of the GSA field. **a** GSARefDB: A screenshot of the R/shiny interface to GSARefDB, showing the options of searching by year, tool name, paper’s first author, title, type of GSA, and programming language. **b** GSA BenchmarKING: One jupyter notebook containing an R workflow for benchmarking single-sample GSA methods, and one shiny app with the same purpose. Both tools display sensitivity, specificity, and precision plots for all the methods under study. See: https://gsa-central.github.io/gsarefdb.html and https://gsa-central.github.io/benchmarKING.html

The information was classified into: (i) Generic methods/software/platforms, usually dealing with mRNA datasets; (ii) Reviews/benchmark studies; (iii) Genomic GSA, which includes GSA applied to enrichment of genomic regions (such as those coming from ChIP-seq, SNP, and methylation experiments); and (iv) ncRNA GSA, which includes methods dealing with miRNA and lncRNA datasets. All information in the database was manually extracted from the papers. Numbers of citations were extracted from Google Scholar (https://scholar.google.com). Only methods that associate *omics* data to annotated gene sets were included (see all types of included methods in Fig. 1b). Bioinformatics methods that associate *omics* data to newly generated modules on a biological network were not included. GSARefDB was built both as an excel sheet and as a shiny app (see Fig. 2a).

### Descriptive statistics

Plots of summary statistics were generated using the “ggplot2” R package. The R code is open and can be found at: https://github.com/antonio-mora/paperCode/blob/master/2019_Mora_Popularity_versus_Performance.R

### Popularity rankings

Popularity rankings in GSARefDB are constructed on a per paper basis, not per-method or per-tool. In order to build popularity rankings of methods or tools that are presented in multiple papers, we use the citation count of the most cited paper for that tool.

### Performance study

The scientific validation approaches followed by the top 153 GSA tool papers in our database were manually reviewed (see Additional file 1: Tab 6). Validation, as performance, was defined as the success of a method on getting better scores than rival methods for a specific quantitative property that measures the agreement between the method's output and either empirical data, simulated data, or the output of another method. Our definition of validation does not include:

(i) Examples of application of the method followed by highlighting the reasonableness of the results (without comparing to other methods),
(ii) Arguments (usually statistical) stating that new assumptions are better than old ones, without any comparison to empirical or properly simulated data, or
(iii) Comparisons of capabilities between old and new software (such as implementing other algorithms or databases).

Detailed procedures followed during the benchmark and simulation studies are explained in Additional file 2.

### Construction of performance-measuring software

The “GSA-BenchmarKING” repository (see Fig. 2b and https://gsa-central.github.io/benchmarKING.html) was created to store and share different tools to measure GSA method performance. For a benchmarking software to be accepted into the repository, it should: (i) Be open software (ideally, a jupyter notebook, RStudio notebook, or shiny app); (ii) Have a clear reason for selecting the GSA methods included; for example, because all of them belong to the same type of methods; (iii) Include both a gold standard dataset and options to upload user-selected gold standard datasets; (iv) Include either a list of target pathways linked to the gold standard dataset or disease relevance scores per pathway for the diseases related to the gold standard; (v) Give the user the option of selecting different benchmarking metrics (as a minimum, precision/sensitivity, prioritization, and specificity/FPR); (vi) Possibility of selecting ensemble results; (vii) Possibility of adding new GSA methods to the code in the future.

The “GSARefDB”, “ss-shiny”, and “gr-shiny” apps were built using the “shiny” package. “GSARefDB” and “ss-shiny” were built using R 3.6.2, while “gr-shiny” was built using R 4.0.0. Open code can be accessed at: https://github.com/gsa-central/gsarefdb, https://github.com/mora-lab/ss-shiny, and https://github.com/mora-lab/gr-shiny.

## Supplementary Information

**Additional file 1**. GSA Reference DB.

**Additional file 2**. Detailed Performance Study.

## Data Availability

All datasets generated for this study are included in the supplementary material. Updated versions of the datasets will be available at https://gsa-central.github.io/gsarefdb.html and https://gsa-central.github.io/benchmarKING.html.
