# Supplementary material for: Popularity and performance of bioinformatics software: the case of gene set analysis
Source: BMC Bioinformatics. 2021 Apr 15;22:191. doi: 10.1186/s12859-021-04124-5 (PMC8050894; doi:10.1186/s12859-021-04124-5)
Supplement: Supplementary file 2 — Additional file 2. Detailed Performance Study. [file 12859_2021_4124_MOESM2_ESM.docx]

**"Popularity and performance of bioinformatics software –The case of Gene Set Analysis"**

**Chengshu Xie^1^, Shaurya Jauhari^1^, and Antonio Mora^1^**

**^1^**Joint School of Life Sciences, Guangzhou Medical University and Guangzhou Institutes of Biomedicine and Health - Chinese Academy of Sciences

**Supplementary Material 2 - Detailed Performance Analysis**

**Contents:**

1. Study of independent benchmarks.

2. Study of independent simulations.

3. Comparison of tools with highest popularity and performance.

***1. Study of independent benchmarks:***

References to all independent and comprehensive benchmark and simulation studies found in the Gene Set Analysis (GSA) field were collected in the Supplementary material 1 (tab 2) and their results will be discussed below.

In early benchmark studies, Naeem *et al.* compared 14 over-representation analysis (ORA) and functional class scoring (FCS) tools, and reported that Analysis of Variance (ANOVA) was the best performer, followed by Z-SCORE and Wilcoxon’s rank-sum (WRS) tests [1]. Hung *et al.* compared six tools: Chi-squared, one so-called global test (Hotelling’s T2), and four gene-set statistics for GSEA (Mean, Median, WRS, and WKS), and reported that WRS and WKS were the best performers [2]. However, those studies were focused on early ORA and FCS methods, with the latter measuring performance as the agreement between tools.

The study by Tarca *et al.* [3] is the largest benchmark study in the field according to our GSARefDB database records. They compared 16 GSA methods split into two categories and evaluated performance on a benchmark dataset made of disease datasets associated with a particular target gene set [3] (for details on the procedure see the main article, “Performance” section, or Figure 1 of reference [3]). The overall ranking of tools is shown in Table 1.

**Supplementary Table 1.** Overall ranking of tools, according to Tarca *et al.* (2013).

| **Category 1 (low False Positive Rate)** | **Category 2 (high FPR)** |
| --- | --- |
| 1. PLAGE,  2. GLOBALTEST,  3. PADOG,  4. ORA,  5. SAFE,  6. SIGPATHWAY-Q2,  7, GSA,  8. SSGSEA,  9. Z-score,  10. GSEA,  11. GSVA,  12. CAMERA. | 1. MRGSE,  2. GSEAP,  3. GAGE,  4, SIGPATHWAY-Q1. |

The authors concluded that the best methods for prioritization (those locating the target gene set in the best position of the final ranking) were different from the most sensitive methods (those producing lower significance value for the target gene set). The most sensitive method was Globaltest [4], followed by PLAGE [5] and GAGE [6] (the worst: GSEA, GSVA, and CAMERA). The best prioritization was offered by PADOG [7], ORA, and MRGSE (worst: CAMERA, SIGPATHWAY-Q1, GSVA). The lowest false positive rate (FPR) appeared in CAMERA [8], SIGPATHWAY-Q2 [9], and Z-SCORE [10] (worst: MRGSE, GSEAP, SIGPATHWAY-Q1, and GAGE). The best compromise considering sensitivity and prioritization was achieved using PLAGE, followed by Globaltest and PADOG, among the methods with a good FPR. Surprisingly, ORA was the best overall method for large gene sets. The study also found that some performance claims from the validation procedures of specific papers couldn’t be replicated; for example, GSVA’s claim to outperform Z-SCORE, PLAGE, and SSGSEA, or GAGE’s claim to outperform GSEA.

Other studies compared Pathway-Topology (PT) tools to popular ORA and FCS tools. Bayerlova *et al.* [11] examined one ORA method (Fisher's exact test), two FCS methods (WRS and Kolmogorov-Smirnov), and four PT methods (SPIA [12], CePa ORA, CePa FCS, and PathNet [13]), using a benchmark of 36 datasets and two simulation studies. They reported that PT and non-PT methods have similar results in benchmark studies, while differences can be found through simulations. The best methods in the benchmark were CePa GSA and PathNet (for sensitivity) and PathNet and WRS (for prioritization). In a simulation study using overlapping KEGG pathways, none of the PT methods outperformed ORA and FCS, while, in a simulation using non-overlapping pathways, all PT methods had higher accuracy than both ORA and FCS, and higher sensitivity than ORA. They concluded that PT-based methods only outperform FCS methods when there are no overlapping pathways, although it is important to note that PT methods including pathway crosstalk are an active field of research. Jaakkola *et al.* [14], on the other hand, reported that PT tools (SPIA, CePa, and NetGSA) find more results than DAVID and GSEA. Another benchmark study compared Network Interaction (NI) tools to ORA and FCS tools using a version of Tarca's benchmark [15]. The results are that BinoX [16] was the most sensitive and less specific tool, while PADOG [7] was the least sensitive and most specific. The Fisher's test and the EASE method (ORA) had an intermediate performance, with EASE being less sensitive and more specific than Fisher's. In terms of prioritization, PADOG was the best performer.

We can also note that Zyla *et al.* benchmarked 16 different ranking metrics to be used inside GSEA [17]. The main result of the study was that the Moderated Welch Test has the best sensitivity while the Minimum Significant Difference has the best specificity. In addition, the Baumgartner-Weiss-Schindler test statistic has the best results when the number of non-normally distributed genes is high.

More recently, Lim *et al.* [18] compared 13 single-sample / individualized pathway-activity methods including ssGSEA, SAS, Pathifier, individPath, ESEA, LLR, DART, PADOG, PLAGE, GSVAmax, GSVAdiff, CORG, and PathAct. They measured the performance on three classification tasks: tumor versus normal, survival information, and cancer subtypes. The first task was dominated by ESEA; the second task by Pathifier and SAS; and the third task by SAS and PADOG. However, the authors highlight that the differences in performance were small and, therefore, focus on two other tests: the preservation of the original data structure (best: Pathifier, PLAGE, and SAS) and the robustness to noise (best: ssGSEA), besides the reproducibility between pathway databases (best: Pathifier, SAS, individPath). Combining the performance in the different tests, Pathifier and SAS are the best methods, followed by PLAGE and individPath.

Nguyen *et al.* [19] compared five PT methods (SPIA, ROntoTools, CePaORA, CePaGSA, PathNet) against eight non-PT methods (Fisher’s exact test, WebGestalt, GOstats, KS, WRS, GSEA, GSA, and PADOG). The authors used a combination of approaches: Data from known diseases that are used as target pathways to be identified, data from mouse KOs used to identify target pathways as those with the KO gene, and a simulation using randomly selected healthy pathways to detect bias under the null hypothesis. Regarding the disease approach (15 different diseases, with 5 datasets per disease), the best prioritization (median rank) of the target pathways was achieved by PADOG, CePaGSA, ROntoTools, and PathNet, while the best prioritization (median p-value) was obtained by CePaGSA, PADOG, and CePaORA. Regarding using mouse KOs, ROntoTools had the highest AUC, followed by GSEA and SPIA; however, this comparison left out some of the previous best performers such as CePaGSA, CePaORA, and PathNet, which didn’t support mouse pathways, so the comparison is limited. The authors also use the questionable procedure of averaging the results of each group of methods to claim that PT methods outperform their counterpart.

Ma *et al.* [20] compared one FCS method (CAMERA), five PT methods (Pathway-Express/ROntoTools, SPIA, CePa, PRS, and PathNet), and three NI methods (NetGSA, topologyGSA, DEGraph). The authors measured prioritization (ranking) using both gene expression and metabolomic datasets in their comparisons. They suggest that, for large signaling pathways, most methods have a good performance, with DEGraph being the best, followed by PathNet. On the other hand, NetGSA and DEGraph were the best performers for small biochemical pathways and metabolomic data.

Zyla *et al.* [21] compared nine methods (PLAGE, GLOBALTEST, GSEA, GSVA, CERNO, Wilcoxon GST, GeneSetTest, ORA, and PADOG), through five different metrics: Sensitivity, FPR, prioritization, computational time, and reproducibility. The authors report that PLAGE has the best results in terms of sensitivity, followed by GLOBALTEST, while methods such as GSVA, ORA, and PADOG have low sensitivity. Regarding specificity/FPR, ORA and PADOG showed the best results. PADOG had also the best results in terms of prioritization (while ORA and GSVA performed poorly). However, in terms of reproducibility, CERNO happened to be the best performer, followed by GeneSetTest, while PLAGE and GLOBALTEST performed poorly. That motivated the idea of reproducibility being an important metric to use together with sensitivity, and methods such as PLAGE and GLOBALTEST might be finding many target pathways but such results could be inconsistent between similar datasets.

Finally, Geistlinger *et al.* [22] compared ten methods and found that ROAST and GSVA were the best performers among those methods assuming a self-contained hypothesis, while ORA and PADOG were the best among those assuming a competitive hypothesis. In terms of runtime, CAMERA, GLOBALTEST, and ORA were the best performers, while GSA, PADOG, and, especially, GSEA, were the worst.

The previous review has been used to support the claim that benchmark studies are few, small, and inconsistent, but, as a constant, no recent benchmark study agrees with the popularity ranking.

***2. Study of independent simulations:***

In addition to benchmark studies, simulation studies also deserve our attention [23]. One variety of simulations adds noise to a dataset and evaluates the performance of the algorithms in the presence of such noise to assess robustness. In a second variety, a dataset is generated following a statistical distribution with certain desired properties, which should either be discovered or rejected by the method. As an example of the second variety, Ackermann and Strimmer [24] built eight datasets of 20 genes each, with values extracted from a multivariate normal distribution with variances equal to 1 and different values of differential expression (positive or negative mean) and correlation; besides that, one dataset of 20 genes with no differential expression (mean equal zero) and no correlation, was built to be used as a negative control. The nine datasets were used as input to compare multiple FCS methods, including seven gene-level statistics (fold change, signal-to-noise ratio, t-statistic, regression coefficient, log-likelihood ratio, correlation coefficient), seven gene-set statistics (Wilcoxon rank test, maxmean statistic, mean, median, enrichment score, sign test, conditional FDR), and three procedures of significance assessment. For each case, the authors compared the sensitivity (percentage of significant p-values) between methods. In the end, they concluded that the choice of a gene-level score is less important than the gene-set statistics. Regarding gene set statistics, all of the options rejected the simulated negative control, while the other eight datasets were compared in terms of sensitivity; in general, the *Mean* test was the most sensitive, followed by the *Median* and *WRS* tests, while GSEA’s enrichment score (ES) was the least sensitive. Their study also included the evaluation of three global tests (which do not compute a gene-level score) and two experimental datasets, which we will not discuss here, but it is important to highlight that the results of the experimental datasets were inconsistent with each other and inconsistent with the results of the simulated data.

A different strategy is to take known gene sets, split them into two parts, and then check how well does a given tool perform using one half as query set and finding the other half as “gold standard” set; such method provides us with a measure of the true positives rate. A randomized version of the bisected gene set list is built by switching each gene with a gene that has a similar degree in the network; using the GSA tools on such a version gives us their false positive rate. This strategy has been useful to prove the advantages of Network Interaction methods for non-overlapping sets, and examples of its usage are RIDDLE [25], CrossTalkZ [26], and BinoX [16].

Nguyen *et al.* [19] implemented a simulation to detect bias under the null hypothesis. In this case, healthy datasets were used (2000 times, datasets randomly selected each time). P-values would be expected to be uniformly distributed between 0 and 1, but most GSA methods proved to be biased towards 0 (consistently lower p-values) or 1 (consistently higher p-values), or both (bimodal). GSEA and PathNet produced no bias towards 0, while GSEA, CePaGSA, WRS, and Fisher’s exact test produced no bias towards 1.

Currently, there is an R package to generate simulated data for GSA purposes called simPATHy [27], which is based on probabilistic graphical models.

***3. Comparison of tools with highest popularity and performance:***

**Supplementary Table 2.** Comparison of tools with highest popularity and performance.

| **Tool** | **Category** | **High Popularity** | **High Performance** |
| --- | --- | --- | --- |
| DAVID / EASE | ORA | YES | YES |
| Hypergeometric / ClueGO / BiNGO / GOseq / clusterProfiler / KOBAS / WEGO | ORA | YES | YES |
| GSEA | FCS | YES | NO |
| PADOG | FCS | NO | YES |
| GLOBALTEST | FCS | NO | YES |
| ROAST | FCS | NO | YES |
| PLAGE | SS | NO | YES |
| Pathifier | SS | NO | YES |
| GSVA | SS | NO | YES |
| PathNet | PT | NO | YES |
| CePa | PT | NO | YES |
| DEGraph | PT | NO | YES |
| SPIA | PT | NO | YES |
| NetGSA | NI | NO | YES |

Only ORA tools belong to both the most popular and the best performing tools. In all other cases, there seems to be an inverse relationship between popularity and performance. "High Popularity" is defined as belonging to the top most cited papers. "High Performance" is defined as being reported as one of the best performing tools under analysis in any of the 10 studied benchmarks.

**References:**

1. Naeem, H., et al., *Rigorous assessment of gene set enrichment tests.* Bioinformatics, 2012. **28**(11): p. 1480-6.

2. Hung, J.H., et al., *Gene set enrichment analysis: performance evaluation and usage guidelines.* Brief Bioinform, 2012. **13**(3): p. 281-91.

3. Tarca, A.L., G. Bhatti, and R. Romero, *A comparison of gene set analysis methods in terms of sensitivity, prioritization and specificity.* PLoS One, 2013. **8**(11): p. e79217.

4. Goeman, J.J., et al., *A global test for groups of genes: testing association with a clinical outcome.* Bioinformatics, 2004. **20**(1): p. 93-9.

5. Tomfohr, J., J. Lu, and T.B. Kepler, *Pathway level analysis of gene expression using singular value decomposition.* BMC Bioinformatics, 2005. **6**: p. 225.

6. Luo, W., et al., *GAGE: generally applicable gene set enrichment for pathway analysis.* BMC Bioinformatics, 2009. **10**: p. 161.

7. Tarca, A.L., et al., *Down-weighting overlapping genes improves gene set analysis.* BMC Bioinformatics, 2012. **13**: p. 136.

8. Wu, D. and G.K. Smyth, *Camera: a competitive gene set test accounting for inter-gene correlation.* Nucleic Acids Res, 2012. **40**(17): p. e133.

9. Tian, L., et al., *Discovering statistically significant pathways in expression profiling studies.* Proc Natl Acad Sci U S A, 2005. **102**(38): p. 13544-9.

10. Lee, E., et al., *Inferring pathway activity toward precise disease classification.* PLoS Comput Biol, 2008. **4**(11): p. e1000217.

11. Bayerlova, M., et al., *Comparative study on gene set and pathway topology-based enrichment methods.* BMC Bioinformatics, 2015. **16**: p. 334.

12. Tarca, A.L., et al., *A novel signaling pathway impact analysis.* Bioinformatics, 2009. **25**(1): p. 75-82.

13. Dutta, B., A. Wallqvist, and J. Reifman, *PathNet: a tool for pathway analysis using topological information.* Source Code Biol Med, 2012. **7**(1): p. 10.

14. Jaakkola, M.K. and L.L. Elo, *Empirical comparison of structure-based pathway methods.* Brief Bioinform, 2016. **17**(2): p. 336-45.

15. De Meyer, S., *Assessing the performance of network crosstalk analysis combined with clustering*, in *VIB - Department Plant Systems Biology*. 2016, Universiteit Gent.

16. Ogris, C., et al., *A novel method for crosstalk analysis of biological networks: improving accuracy of pathway annotation.* Nucleic Acids Res, 2017. **45**(2): p. e8.

17. Zyla, J., et al., *Ranking metrics in gene set enrichment analysis: do they matter?* BMC Bioinformatics, 2017. **18**(1): p. 256.

18. Lim, S., et al., *Comprehensive and critical evaluation of individualized pathway activity measurement tools on pan-cancer data.* Brief Bioinform, 2018.

19. Nguyen, T.M., et al., *Identifying significantly impacted pathways: a comprehensive review and assessment.* Genome Biol, 2019. **20**(1): p. 203.

20. Ma, J., A. Shojaie, and G. Michailidis, *A comparative study of topology-based pathway enrichment analysis methods.* BMC Bioinformatics, 2019. **20**(1): p. 546.

21. Zyla, J., et al., *Gene set enrichment for reproducible science: comparison of CERNO and eight other algorithms.* Bioinformatics, 2019. **35**(24): p. 5146-5154.

22. Geistlinger, L., et al., *Toward a gold standard for benchmarking gene set enrichment analysis.* Brief Bioinform, 2020.

23. Giannoulatou, E., et al., *Verification and validation of bioinformatics software without a gold standard: a case study of BWA and Bowtie.* BMC Bioinformatics, 2014. **15 Suppl 16**: p. S15.

24. Ackermann, M. and K. Strimmer, *A general modular framework for gene set enrichment analysis.* BMC Bioinformatics, 2009. **10**: p. 47.

25. Wang, P.I., et al., *RIDDLE: reflective diffusion and local extension reveal functional associations for unannotated gene sets via proximity in a gene network.* Genome Biol, 2012. **13**(12): p. R125.

26. McCormack, T., et al., *Statistical assessment of crosstalk enrichment between gene groups in biological networks.* PLoS One, 2013. **8**(1): p. e54945.

27. Salviato, E., et al., *simPATHy: a new method for simulating data from perturbed biological PATHways.* Bioinformatics, 2017. **33**(3): p. 456-457.
